# Editorial announcement regarding title change of *Chinese Journal of Cancer* to *Cancer Communications*

**DOI:** 10.1186/s40880-018-0270-7

**Published:** 2018-03-09

**Authors:** Rui-Hua Xu

**Affiliations:** Sun Yat-sen University Cancer Center, Guangzhou, 510060 China

On behalf of the editorial board, I am pleased to announce the title change of *Chinese Journal of Cancer* (*Chin J Cancer*, *CJC*; eISSN, 1944-446X) to *Cancer Communications* (*Cancer Commun*, *CC*; eISSN, 2523-3548). From March 2018, the journal will be published under the new title *Cancer Communications* with the same scope of publication as the former title. *CJC* was launched in 2010 as the official journal of the Chinese Anti-Cancer Association (CACA) and the US Chinese Anti-Cancer Association (USCACA). Under its new name, *Cancer Communications* continues to be sponsored by Sun Yat-sen University Cancer Center [1]. *Cancer Communications*, like its predecessor *CJC* has been since 2014, will be indexed in Medline/PubMed, PubMed Central, Directory of Open Access Journals (DOAJ), Scopus, the Western Pacific Region Index Medicus, CA, and Science Citation Index Expanded (SCIE) continuously.

With an international editorial board and science editors, our journal will continue to publish peer-reviewed, open access, and English-language scientific articles in the areas of basic, clinical, and translational cancer research that aim to improve human health through understanding cancer, preventing cancer onset, reducing cancer mortality, and improving the quality of life of cancer survivors worldwide [2]. Our journal emphasizes timely cancer issues, and under its previous title has published several thematic series such as air pollution and cancer [3], tumor metastasis [4], tumor angiogenesis [5], gastric cancer [6], and the 150 most important questions in cancer research and clinical oncology [7], among others.

The impact of *CJC* in the international arena has been steadily increasing. We are very pleased to see that the historical impact of *CJC* has been markedly augmented in the world. The Journal Citation Report (JCR) Impact Factor (IF) of *CJC* increased from 2.814 in 2015 to 4.111 in 2016.

We are grateful to all the editorial board members from all over the world for their contributions toward improving the journal and in selecting the new journal title. We especially want to thank all of our authors for contributing their wonderful work and all the reviewers for their professional and thoughtful input in helping the journal to achieve its high standards.

The new title, *Cancer Communications*, is a new beginning. I hope that *Cancer Communications* will continue to make fundamental contributions to the field of cancer research and help to strengthen communication and collaboration among oncology researchers all over the world.
